# Uncover the genetic basis of processing quality related traits in common wheat (*Triticum aestivum* L.) using genome-wide association mapping

**DOI:** 10.3389/fpls.2026.1755182

**Published:** 2026-03-03

**Authors:** Quanhao Song, Wenwen Cui, Jiajing Song, Baoyuan Zhou, Liang Chen, Kaijie Xu, Yan Jin

**Affiliations:** 1 Zhumadian Academy of Agricultural Sciences, Henan Provincial Engineering Research Center for Germplasm Improvement and Breeding of Multi-Resistant and High-Efficiency Wheat, Zhumadian, China; 2 Zhumadian Academy of Industry Innovation and Development, Huanghuai University, Zhumadian, China; 3 Institute of Crop Science, Chinese Academy of Agricultural Sciences, Beijing, China; 4 State Key Laboratory for Crop Stress Resistance and High-Efficiency Production, Collage of Agronomy, Northwest A&F University, Yangling, China; 5 Institute of Cotton Research, Chinese Academy of Agricultural Sciences, Anyang, China

**Keywords:** association mapping, common wheat, SDS sedimentation volume (SSV), test weight, water absorption rate

## Abstract

**Introduction:**

Improving wheat processing quality is one of the primary objectives in modern wheat breeding. Among various wheat quality parameters, SDS sedimentation volume (SSV), test weight (TW), and water absorption rate (WAR), significantly influence end-use flour quality. The Huang-Huai Winter Wheat Region (HHWWR) is the largest commercial wheat production region in China, making the breeding of high-quality wheat varieties adapted to this region particularly important.

**Methods:**

In this study, genome-wide association study (GWAS) analysis for grain quality traits were conducted based on 310 wheat varieties collected from HHWWR. The SSV, TW, and WAR were evaluated at Anyang of Henan and Yangling of Shaanxi at the 2022–2023 and 2023–2024 cropping seasons.

**Results and discussion:**

Totally, three stable SSV related loci were detected on chromosomes 1A and 4A, explaining 7.2-9.2% of the phenotypic variation (PVE). Seven stable TW loci were distributed on chromosomes 1A, 1B, 4A, 5A, 6D, and 7B, with PVE ranging from 7.0% to 20.1%. In addition, 5 stable WAR-related loci were found on chromosomes 1A, 3B, 4B, and 4D, and accounting for 7.0-8.7% of PVE. Among these, 5 loci co-localized with known genes or loci, whereas the remaining 10 loci are potentially novel. We further identified several candidate genes involved in various biological pathways in plants, including growth and development, stress responses, metabolic regulation, and signal transduction. Moreover, five Kompetitive Allele-Specific PCR (KASP) markers, *Kasp-SSV-1AS*, *Kasp-SSV-4AL*, *Kasp-TW-1AL*, *Kasp-TW-7BL*, and *Kasp-WAR-1AL*, were developed and validated in 123 common wheat accessions. This study provides stable loci and validated KASP markers, thereby paving the way for the molecular improvement of wheat quality through marker-assisted breeding.

## Introduction

Common wheat (*Triticum aestivum* L.) is a major global staple crop, serving as the primary food source for 35-40% of the world population (He et al., 2011; Guo et al., 2023). With ongoing economic development and rising living standards, consumer demand for high-quality wheat continues to increase. Although wheat quality has improved significantly, it still lags behind the levels in developed countries and falls short of market expectations (Miao et al., 2022; Guo et al., 2023; Khalid et al., 2023; Zahra et al., 2023). Therefore, enhancing grain quality has become an urgent priority in wheat breeding programs (Kiszonas and Morris, 2018; Olaerts and Courtin, 2018; Liu et al., 2024).

Wheat processing quality is predominantly governed by a suite of key physicochemical parameters (He et al., 2011). Key quality parameters include SDS sedimentation value (SSV), test weight (TW) and water absorption rate (WAR). Among these, the SSV serves as a direct proxy for gluten strength; higher SSV translates to dough with greater elasticity and stability, ultimately yielding bread with larger volume and finer crumb structure (Tian et al., 2021; Mohamed et al., 2022; Huang et al., 2024). TW, an indicator of kernel plumpness and density, is crucial for milling efficiency and flour yield, directly impacting economic returns (Tadesse et al., 2015; Li et al., 2019; White et al., 2022). WAR dictates the hydration capacity of flour, fundamentally influencing dough handling properties, processing tolerance, and the texture of end-products like noodles and bread (Guo et al., 2023; Shahi et al., 2024; Khan et al., 2024; Li et al., 2024; Subedi et al., 2024; Chen et al., 2025; Jia et al., 2025). Therefore, concerted improvement of SSV, TW, and WAR is essential for developing wheat varieties that meet the stringent requirements of the modern food industry.

These quality traits are complex and quantitatively inherited, influenced by genotype, environment, and their interactions (Guo et al., 2023). Conventional breeding approaches are often time-consuming and inefficient. Although previous studies have identified quantitative trait loci (QTL) for SSV, TW, and WAR (Reif et al., 2011; Chen et al., 2017; Schulthess et al., 2017), most reported intervals remain broad and rely on low-throughput markers such as SSR, and limiting their utility in breeding (He et al., 2020). Genome-wide association studies (GWAS) offer a powerful approach to elucidate molecular mechanisms. In addition, the development of the Kompetitive Allele-Specific PCR (KASP) markers are essential for advancing high-quality wheat breeding (Bordes et al., 2014; Huang and Han, 2014; Rasheed et al., 2016; Kaur et al., 2020; Tian et al., 2021).

However, the successful application of GWAS in dissecting complex quality traits hinges on high-density, high-quality genotyping. Modern high-throughput SNP arrays, such as the Wheat 90K or 660K arrays, have dramatically improved mapping resolution and power for GWAS in wheat (Tibbs Cortes et al., 2021; Liu et al., 2023, 2024). This advancement allows for the detection of stable, fine-mapped associations that are directly applicable to marker development, a critical step forward from earlier QTL studies. To this end, we employed the Wheat 100K Chip, a high-density SNP array designed based on extensive resequencing data. This platform provides 100K marker regions and 251,215 SNP for a powerful GWAS, enabling us to move beyond broad QTL intervals and identify precise genomic regions associated with SSV, TW, and WAR.

The HHWWR is a major wheat production base in China, recognized for its favorable climate and high yield potential. However, most commercial varieties in this region still lack optimal end-use quality, hindering the industrialization and competitiveness of the local wheat industry. Despite the global importance of these traits, a significant knowledge gap exists. There is a paucity of high-resolution genetic studies simultaneously targeting SSV, TW, and WAR within the context of major production regions like HHWWR. Most previous genetic analyses have either focused on single traits, utilized germplasm from diverse origins not optimized for local adaptation, or lacked the marker density for effective translational breeding.

Therefore, to bridge this gap, we conducted a comprehensive GWAS using the 100K SNP array on a panel of 310 elite, locally-adapted wheat accessions mainly from the HHWWR. Phenotypic data for SSV, TW, and WAR were rigorously collected across multiple environments to account for G×E interactions. Our objectives were to: (1) identify stable and significant MTAs for these key processing quality parameters; (2) propose candidate genes underlying the most stable loci; and (3) develop and validate practical KASP markers to enable MAS for superior wheat quality specifically within the HHWWR breeding pipeline.

## Materials and methods

### Plant materials and field trials

We used 310 elite wheat cultivars for GWAS and an additional 123 varieties to validate the KASPmarkers (**Supplementary Table S1**). The 310 cultivars were primarily from the HHWWR of China, including released cultivars and advanced breeding lines from Henan, Shandong, Anhui, Jiangsu, and Shaanxi provinces. Another 52 winter wheat varieties from Europe were also included in the panel. To verify the usability of KASP markers in the detection of natural varieties, our selected validation population consisted of 123 germplasms. The majority of these germplasms differed from the 310 representative varieties from the Huang-Huai wheat region used in the GWAS analysis population, with only four common materials: Jimai20, Zhoumai22, Zhengmai366, and Zhoumai18.

Field experiments were conducted during the 2022–2023 and 2023–2024 growing seasons at Anyang (Henan) and Yangling (Shaanxi). All trials employed a randomized complete block design with three replications in fields with uniform moderate fertility. Each plot consisted of three 2.0-m-long rows with 25 cm between rows and 10 cm between plants. Seeds were manually sown using the single-seed dibbling method, and field management followed local high-yield practices. After harvest, grains were sun-dried and cleaned to remove impurities and defective kernels. Quality traits (SSV, TW, WAR) were measured using a DA7200 near-infrared analyzer (Swiss-made). Three measurements were taken for each replicate, and the mean values were used for GWAS analysis.

### Genotyping and population structure

Genomic DNA was extracted from young leaf tissue of each accession using a modified cetyltrimethylammonium bromide (CTAB) method (Saghai-Maroof et al., 1984). The 310 cultivars were genotyped using the wheat 100K SNP array (Molbreeding, China). Initial genotype data were filtered to exclude SNPs with >20% missing data or minor allele frequency (MAF)< 0.05. The resulting high-quality SNPs were physically positioned according to the Chinese Spring reference genome (IWGSC v1.0, http://www.wheatgenome.org/). Principal component analysis (PCA) and neighbor-joining (NJ) tree construction were performed using TASSEL v5.0.

### Phenotypic data analysis and heritability estimation

SSV, WAR and TW were assessed across four environments. Data were analyzed with the SAS v9.2. Variance components were estimated via ANOVA (PROC GLM) (Chatzi and Doody, 2023), and phenotypic correlations were computed (PROC CORR). Broad-sense heritability $\left({H_{\text{b}}}^{2}\right)$ was calculated as ${H_{\text{b}}}^{2}={\sigma_{\text{g}}}^{2}/({\sigma_{\text{g}}}^{2}+{\sigma_{\text{ge}}}^{2}/\text{e}+{\sigma_{\epsilon }}^{2}/\text{re})$, where ${\sigma_{\text{g}}}^{2}$, ${\sigma_{\text{ge}}}^{2}$, and ${\sigma_{\epsilon }}^{2}$ are the variances for genotype, genotype-by-environment interaction, and residual error, respectively; e and r refer to environment and replicate counts (Hühn, 1975). T-tests were applied to assess trait differences across marker genotypes, using both per-environment and averaged phenotypic values (Pandis, 2016).

### Association analysis and candidate gene identification

The population structure among the 310 wheat accessions was assessed using PCA and phylogenetic analysis. To reduce false-positive associations in GWAS, we employed a mixed linear model (MLM) in Tassel v5.0, incorporating both PCA and kinship matrix as covariates. In this study, the Bonferroni-Holm correction for multiple testing (α = 0.05) was overly conservative, resulting in no significant MTAs. Therefore, markers with an adjusted –log_10_(*P*-value) ≥ 3.0 were considered statistically significant. Results were visualized as Manhattan and Q-Q plots using the CMplot package in R.

For candidate gene identification, we examined genomic regions extending ± 3.0 Mb from each peak SNP position in the Chinese Spring reference genome (IWGSC v1.0, http://www.wheatgenome.org/). The initial gene list was filtered to remove entries annotated as hypothetical proteins, transposon-related, or retrotransposon-associated proteins. Remaining candidates were evaluated based on functional annotations, with particular attention to known gene families linked to grain quality traits, such as the NAC family. Finally, we analyzed expression patterns of the candidate genes using the public wheat gene expression database (http://wheat-expression.com/).

### KASP marker design and validation

For stable and major-effect loci consistently identified across multiple environments, flanking SNP markers were converted into KASP assays. Primer design was conducted using the online tool PolyMarker, which generated two allele-specific forward primers (each labeled with distinct fluorescent dyes, FAM or HEX) and one common reverse primer. PCR amplification was performed in a 4 µL reaction system containing 2.0 µL of Master Mix, 0.048 µL of primer mix, and 1.952 µL of template DNA (50 ng/µL). The thermal cycling protocol comprised an initial denaturation at 94 °C for 15 min, followed by 10 touchdown cycles of 94 °C for 20 s and 63-55 °C for 60 s (decreasing by 1 °C per cycle), and then 32 additional cycles of 94 °C for 20 s and 55 °C for 60 s. Endpoint fluorescence was measured using a PHERAstarplus plate reader, and genotype calling was automatically performed using KlusterCaller v3.4 software (LGC Group). All developed KASP markers were further validated in a panel of 123 wheat varieties, mainly consisting of elite cultivars and advanced breeding lines from the Huang-Huai Winter Wheat Region, to confirm their genetic effects and applicability in molecular breeding.

## Results

### Phenotypic evaluation

**Figure 1:**
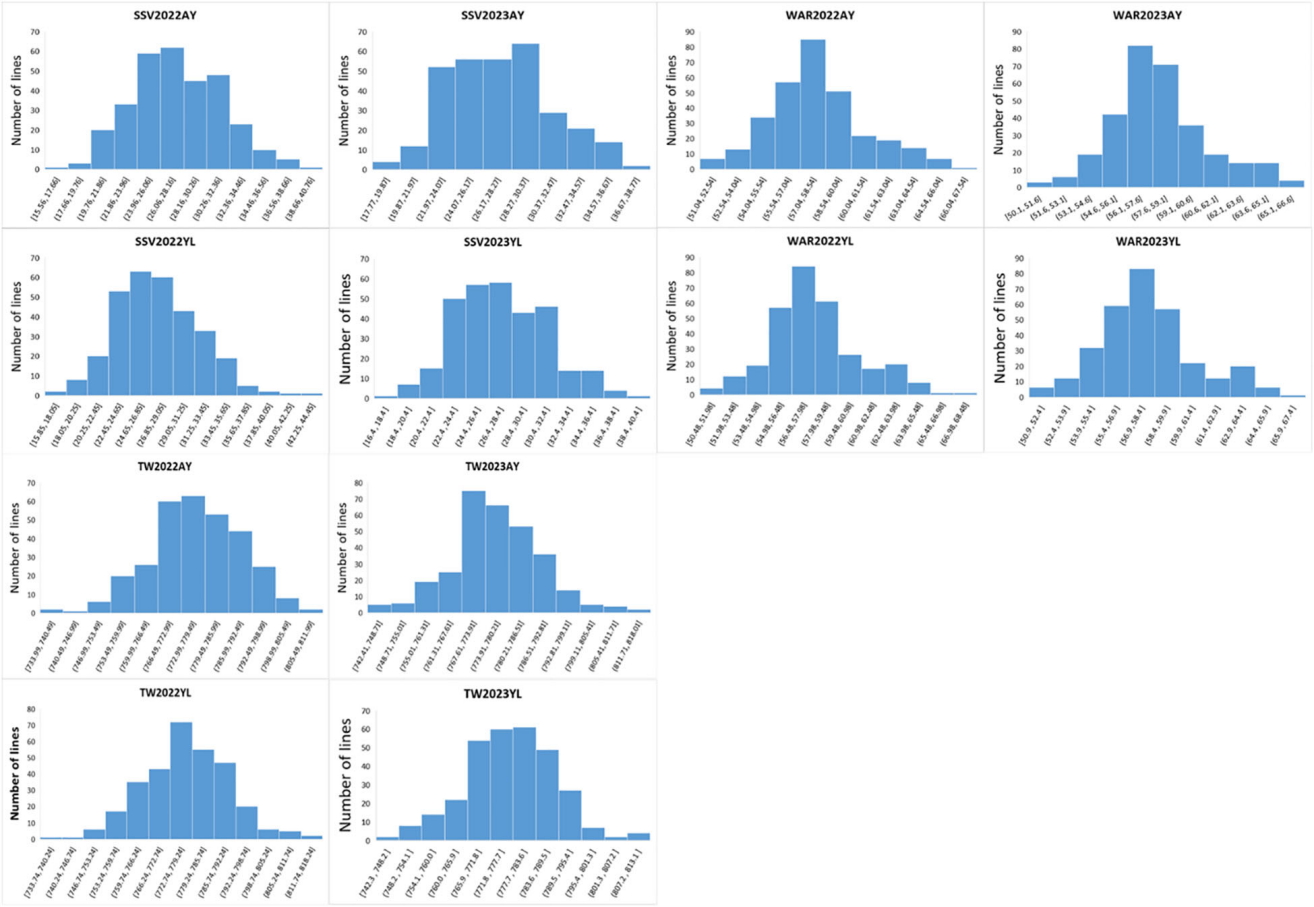
The distribution of the SSV, WAR and TW in the 310 wheat accessions SSV, SDS sedimentation volume; TW, Test weight; WAR, Water absorbing rate.

SSV, TW, and WAR exhibited continuous variation across the four environments. Among the 310 wheat accessions, SSV ranged from 16.4 mL to 39.4 mL, with a mean of 27.5 mL, a standard deviation of 3.9 mL, and a coefficient of variation (CV) of 14.3%. TW varied between 742.3 g/L and 811.7 g/L, averaging 777.1 g/L with a standard deviation of 11.5 g/L and a CV of 1.5%. WAR showed values from 50.9% to 67.3%, with a mean of 58.0%, a standard deviation of 2.8%, and a CV of 4.9% (**Table 1**; **Figure 1**; **Supplementary Table S1**). This substantial phenotypic diversity indicated that the association panel was suitable for genome-wide association analysis. ANOVA revealed highly significant effects (*P*< 0.001) of genotype (G), environment (E), and their interaction (G × E) for all three traits (**Table 1**; **Figure 1**). The *H*_b_² estimates were 0.63 for SSV, 0.59 for TW, and 0.62 for WAR, indicating that genetic factors play a major role in phenotypic variation and supporting the feasibility of association mapping (**Supplementary Table S3**).

**Table 1:** The summary of the SSV, TW and WAR in the 310 winter wheat accessions.

Trait	SSV (mL)	TW (g/L)	WAR (%)
Max	39.4	811.7	67.3
Min	16.4	742.3	50.9
Average	27.5	777.1	58.0
Standard deviation	3.9	11.5	2.8
Coefficient of variation	0.143	0.015	0.049

SSV, SDS sedimentation volume; WAR, Water absorbing rate; TW, Test weight.

### Genotyping and population structure

**Figure 2:**
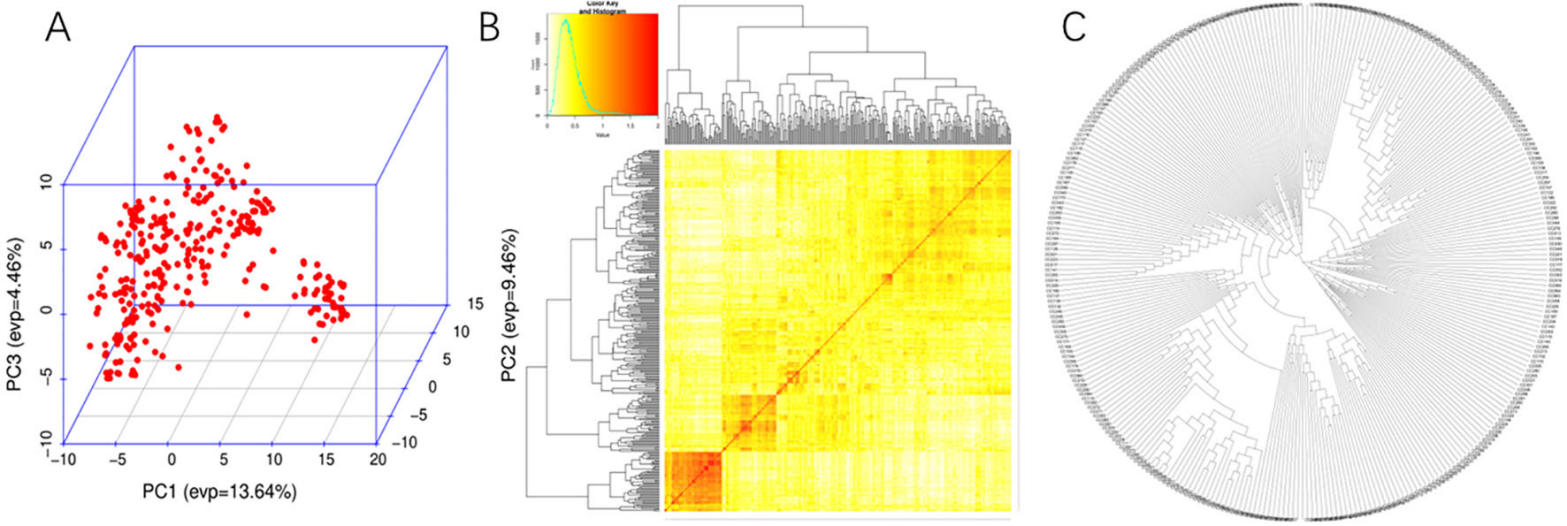
The **(A)** PCA, **(B)** NJ-tree and **(C)** kinship for the 310 wheat accessions.

Genotyping of all 310 wheat accessions was performed using the wheat 100K SNP array aligned to the Chinese Spring reference genome (IWGSC v1.0). After quality control, 108,836 high-quality SNPs were retained for GWAS (**Supplementary Table S4**). These markers spanned a total physical distance of 14,223.3 Mb across the genome, yielding an average density of 7.7 markers/Mb (**Supplementary Table S4**). Population structure analysis revealed four distinct subgroups: Subgroup 1 (n = 104)predominantly comprised accessions from Henan and Shandong Provinces; Subgroup 2 (n = 89) included accessions from southern Henan and Anhui; Subgroup 3 (n = 67) consisted of accessions from northern Henan and Shaanxi; and Subgroup 4 (n = 52) contained accessions of European origin. Linkage disequilibrium (LD) decay in this Chinese wheat panel occurred at 3.0–5.0 Mb, confirming sufficient marker density for further GWAS analysis (Liu et al., 2017; **Supplementary Table S1**, **Figure 2**).

### Association analysis for grain quality related traits

**Figure 3:**
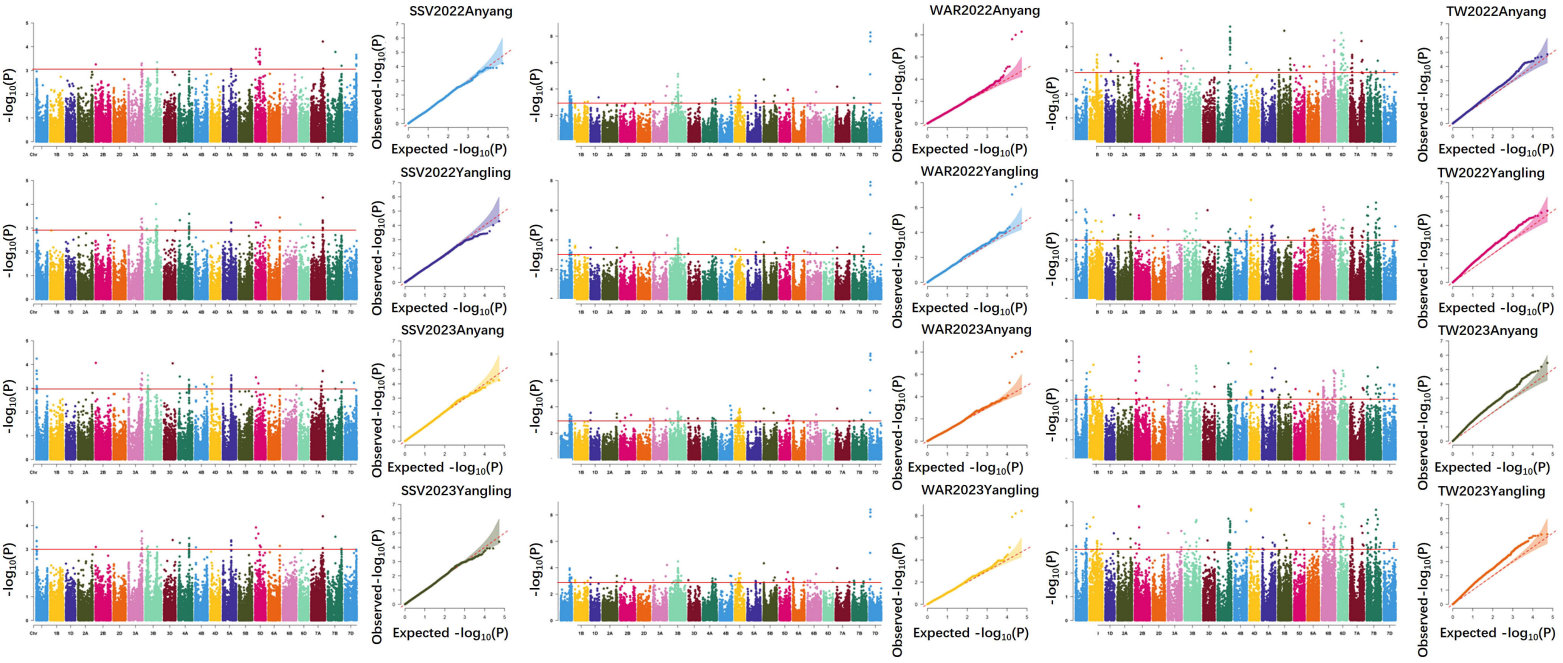
Manhattan and Q-Q plot for SSV, WAR and TW in the 310 wheat accessions.

GWAS was conducted for SSV, TW and WAR based on the 310 wheat accessions and 15 stable loci were identified. For SSV, three stable loci were mapped on chromosomes 1A and 4A. Among these, *QSSV.zaas-1AS* located at 26.4-27.9 Mb on chromosome 1A accounted for 7.2-9.2% of the PVE. Another locus, *QSSV.zaas-249-4AS* located on chromosome arm 4AS (85.8 Mb), exhibited 8.3-9.0% of the PVE. *QSSV.zaas-4AL* located on chromosome arm 4AL (578.8-581.8 Mb), demonstrated stable effects and explained 7.2-8.8% of the PVE (**Table 2**; **Figure 3**).

**Table 2:** The loci for SSV, TW and WAR identified in the 310 winter wheat accessions by GWAS.

Name ^a^	Env. ^b^	Chr. ^c^	Position (Mb)	*P*-value	R^2^ (%)
*QSSV.zaas-1AS*	E1, E2	1A	26.4-27.9	1.62E-04~7.40E-04	7.2-9.2
*QSSV.zaas-4AL*	E1, E3	4A	578.8-581.8	5.98E-04~7.43E-04	7.2-8.8
*QSSV.zaas-4AS*	E2, E3	4A	85.8-85.8	2.66E-04~3.96E-04	8.3-9.0
*QTW.zaas-1AL*	E2, E3	1A	582.0-582.0	1.17E-04~5.54E-04	7.5-9.2
*QTW.zaas-1BS*	E2, E3	1B	26.0-26.0	2.24E-05~8.05E-04	13.2-20.1
*QTW.zaas-4AL1*	E1, E2	4A	578.8-581.8	5.98E-04~7.43E-04	7.2-8.3
*QTW.zaas-4AL2*	E1, E2, E3	4A	678.5-685.7	5.31E-05~7.22E-04	7.3-9.9
*QTW.zaas-5AL*	E2, E3	5A	554.1-569.7	4.49E-05~9.72E-04	7.0-10.7
*QTW.zaas-6DS*	E1, E2, E3	6D	180.1-180.1	1.82E-05~1.93E-04	8.7-11.1
*QTW.zaas-7BL*	E1, E2	7B	493.2-498.7	3.88E-04~7.09E-04	7.3-7.9
*QWAR.zaas-1AL*	E1, E2, E3	1A	477.4-477.4	3.03E-04~7.19E-04	7.3-8.6
*QWAR.zaas-3BL*	E2, E3	3B	454.1-454.1	5.30E-04~8.16E-04	7.2-7.6
*QWAR.zaas-4BL*	E1, E2	4B	623.8-623.8	1.86E-04~9.95E-04	7.0-8.7
*QWAR.zaas-4DS1*	E2, E3	4D	38.4-38.4	3.59E-04~9.95E-04	7.1-8.0
*QWAR.zaas-4DS2*	E1, E2	4D	208.5-208.5	5.73E-04~9.95E-04	7.1-7.6

a SSV, SDS sedimentation volume; TW, Test weight; WAR, Water absorbing rate

b Env., Environment

c Chr., Chromosome; E1, 2022Anyang; E2, 2023Anyang; E3, 2022Yangling; E4, 2023Yangling.

In total, 7 loci were identified for TW on chromosome 1A, 1B, 4A (two loci), 5A, 6D and 7B from the 310 wheat accessions from HHWWR by GWAS. The most substantial effect was attributed to *QTW.zaas-1BS*, located on 26.0 Mb of chromosome 1B with PVE of 13.2-20.1%. Other significant loci for TW were detected at *QTW.zaas-1AL* at 582.0 Mb on chromosome 1A with PVE of 7.5-9.2%; *QTW.zaas-4AL* of 678.5-685.7 Mb on chromosome 4A with PVE from 7.3% to 9.9%. In addition, another locus on chromosome 4A (678.5-685.7 Mb), named as *QTW.zaas-4AL2* were identified with PVE from 7.2% to 8.3%. In addition, a stable locus was identified on chromosomes 5A, e.g. *QTW.zaas-5AL* located at 554.1-569.7 Mb with PVE of 7.0-10.7%. Two significant loci for TW were identified on chromosomes 6D and 7B. The locus *QTW.zaas-6DS* on 180.1 Mb at chromosome 6D with 8.7-11.1% of the PVE, whereas the *QTW.zaas-7BL* on chromosome 7B (493.2-498.7 Mb) accounted for 7.3-7.9%.

Five QTL for WAR were identified on chromosome 1A, 3B, 4B and 4D (two loci). One stable QTL, *QWAR.zaas-1AL* located on 477.4 Mb of chromosome 1A, with 7.3% to 8.6% of the PVE, while another stable locus, *QWAR.zaas-3BL* on chromosome 3B (454.1 Mb) and accounted for 7.2-7.6% of PVE. Another notable locus, *QWAR.zaas-4BL* at the 623.8 Mb of chromosome 4B, with 7.0-8.7% of the PVE. Furthermore, two distinct QTL on the short arm of chromosome 4D, e.g. *QWAR.zaas-4DS.1* located at 38.4 Mb with PVE of 7.1-8.0%; and *QWAR.zaas-4DS.2* located on 208.5 Mb with PVE from 7.1% to 7.6% (**Table 2**; **Figure 3**). In addition, overlapping QTL regions on chromosomes 4A for SSV (*QSSV.zaas-4AL*) and TW (*QTW.zaas-4AL1*) suggest possible pleiotropy or tight linkage genes. These overlapping intervals provide valuable markers for MAS in wheat breeding aimed at improving grain quality and processing characteristics (**Table 2**; **Figure 3**).

### Candidate gene analysis

**Figure 4:**
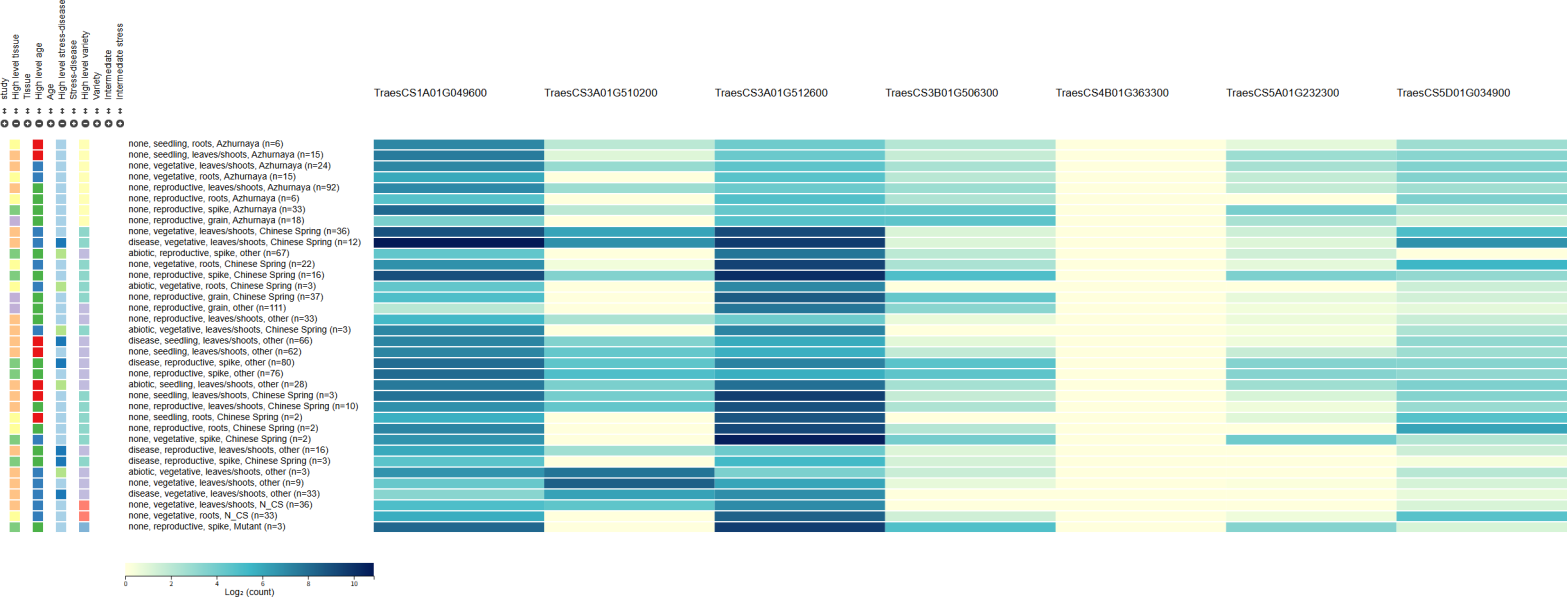
Expression pattern for the candidate genes from public database (http://wheat-expression.com/).

Totally, 15 candidate genes associated with SSV, TW and WAR in common wheat were identified by gene annotation and expression public database (**Table 3**; **Figure 4**). For SSV, candidate genes comprised regulatory proteins and enzymes, including MADS-box transcription factor (*TraesCS1A01G044900*), a beta-1,3-galactosyltransferase-like protein (*TraesCS1A01G047000*), E3 ubiquitin-protein ligase (*TraesCS4A01G083500*), and a zinc finger protein VAR3 (*TraesCS4A01G267600*). For WAR, candidate gene list featured transporters and hydrolases, such as auxin influx transporter (*TraesCS1A01G278400*), beta-glucosidase (*TraesCS1A01G279000*), MYB-related transcription factor (*TraesCS3B01G281500*), ABC transporter (*TraesCS4B01G331400*), zinc finger protein DAYSLEEPER (*TraesCS4D01G062600*), and an E3 ubiquitin-protein ligase (*TraesCS4D01G155700*). For TW, candidates were dominated by signaling components, including ethylene-responsive transcription factors (*TraesCS4A01G412200*, *TraesCS5A01G371300*), serine/threonine-protein kinases (*TraesCS4D01G059300*, *TraesCS5A01G350800*) and ABC transporter (*TraesCS7B01G271500*) (**Supplementary Table S5**).

**Table 3:** The candidate genes for the loci of grain quality identified in the 310 winter wheat accessions by GWAS.

QTL^a^	Candidate	Chromosome	Position (Mb)^b^	Annotation
*QSSV.zaas-1AS*	*TraesCS1A01G044900*	1A	25.7	MADS-box transcription factor
*QSSV.zaas-1AS*	*TraesCS1A01G047000*	1A	26.8	Beta-1,3-galactosyltransferase-like protein
*QWAR.zaas-1AL*	*TraesCS1A01G278400*	1A	474.2	Auxin influx transporter
*QWAR.zaas-1AL*	*TraesCS1A01G279000*	1A	474.6	Beta-glucosidase
*QWAR.zaas-3BL*	*TraesCS3B01G281500*	3B	452.1	MYB-related transcription factor
*QSSV.zaas-4AS*	*TraesCS4A01G083500*	4A	87.4	E3 ubiquitin-protein ligase
*QSSV.zaas-4AL*	*TraesCS4A01G267600*	4A	579.9	Zinc finger protein
*QTW.zaas-4AL2*	*TraesCS4A01G412200*	4A	684.2	Ethylene-responsive transcription factor
*QWAR.zaas-4BL*	*TraesCS4B01G331400*	4B	621.9	ABC transporter family protein
*QWAR.zaas-4DS1*	*TraesCS4D01G059300*	4D	35.2	Serine/threonine-protein kinase
*QWAR.zaas-4DS1*	*TraesCS4D01G062600*	4D	38.4	Zinc finger BED domain-containing protein
*QWAR.zaas-4DS2*	*TraesCS4D01G155700*	4D	208.4	E3 ubiquitin-protein ligase
*QTW.zaas-5AL*	*TraesCS5A01G350800*	5A	553.5	Serine/threonine-protein kinase
*QTW.zaas-5AL*	*TraesCS5A01G371300*	5A	570.2	Ethylene-responsive transcription factor
*QTW.zaas-7BL*	*TraesCS7B01G271500*	7B	498.3	ABC transporter family protein
*QTW.zaas-7BL*	*TraesCS7B01G272300*	7B	499.3	Ethylene-responsive transcription factor

a SSV, SDS sedimentation volume; TW, Test weight; WAR, Water absorbing rate;

b Physical from the IWGSC V1.0.

### KASP marker development and validation

**Figure 5:**
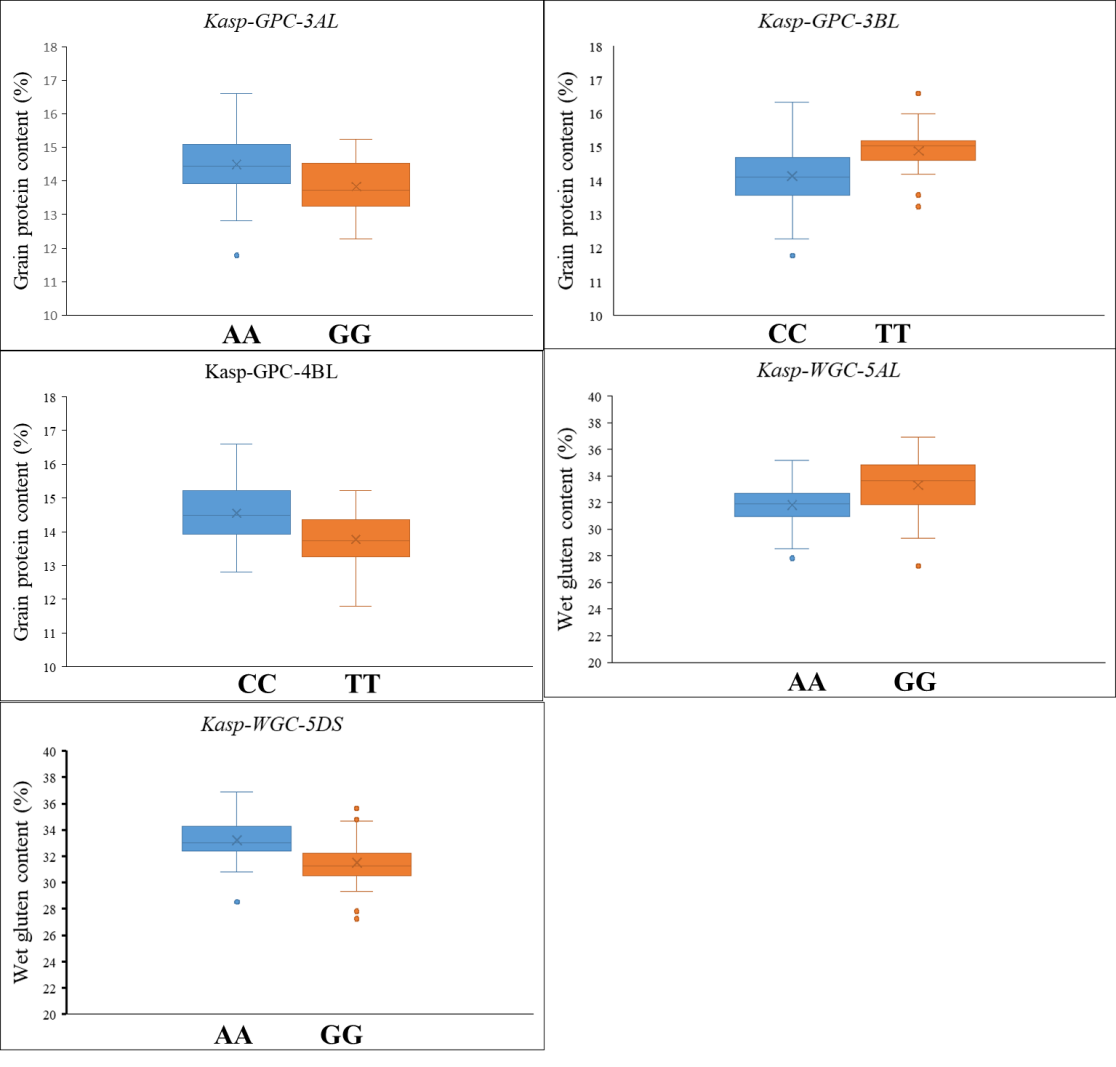
The KASP markers validated in another 123 diverse panel. GPC, grain protein content; WGC, wet grain content.

All QTL were employed in the development of KASP markers. To validate the efficacy of the developed KASP markers, a diverse panel of 123 cultivars was employed. A set of five KASP markers was successfully developed and validated, including *Kasp-SSV-1AS* for *QSSV.zaas-1AS* at 24.2 Mb on chromosome 1A, *Kasp-SSV-4AL* for *QSSV.zaas-4AL* at 576.8 Mb on chromosome 4A, *Kasp-TW-1AL* for *QTW.zaas-1AL* at 581.8 Mb on chromosome 1A, *Kasp-TW-7BL* for *QTW.zaas-7BL* at 591.4 Mb on chromosome 7B, and *Kasp-WAR1-1AL* for *QWAR.zaas-1AL* at 473.6 Mb on chromosome 1A (**Table 4**). Association analysis between marker alleles and phenotypic data revealed significant effects. For *Kasp-SSV-1AS*, accessions with the CC allele (63.4%) exhibited significantly higher mean SSV (31.6 mL) than those with AA allele (36.6%, 29.2 mL; *p<* 0.05). Conversely, for *Kasp-SSV-4AL*, AA allele (57.7%) was associated with a higher SSV (30.6 mL) compared to GG allele (17.1%, 28.5 mL; *p*< 0.05). Regarding TW, CC allele (51.2%) of *Kasp-TW-1AL* corresponded to lower TW (799.1 g/L) relative to TT allele (48.8%, 810.7 g/L; *p<* 0.05). For *Kasp-TW-7BL*, AA allele (29.3%) was identified as favorable, conferring a higher TW (809.8 g/L) than GG allele (45.5%, 797.9 g/L; *p* = 0.05). Similarly, for *Kasp-WAR1-1AL*, AA allele (52.0%) was associated with a higher WAR (61.0%) compared to the GG allele (47.2%, 59.6%; *p* = 0.05) (**Table 5**; **Supplementary Table S6**; **Figure 5**).

**Table 4:** The KASP marker details for SSV, TW and WAR.

Kasp marker	QTL	Chromosome	Position (Mb)	FAM	HEX	Common
*Kasp-SSV-1AS*	*QSSV.zaas-1AS*	1A	24.2	tgacgtcctggacaatgtct	tgacgtcctggacaatgtcg	aatctgggcggcaagacg
*Kasp-SSV-4AL*	*QSSV.zaas-4AL*	4A	576.7	ggcagttaattgtcatcacctca	ggcagttaattgtcatcacctcg	tcaagagggcacatttgagtta
*Kasp-TW-1AL*	*QTW.zaas-1AL*	1A	581.8	gcgagactatgaggtgcttt	gcgagactatgaggtgcttc	ctctgcaacctccgtgtca
*Kasp-TW-7BL*	*QTW.zaas-7BL*	7B	491.4	gtgaccctgaacctcctgaaa	gtgaccctgaacctcctgaag	agtaacaagtcacaggggtttaa
*Kasp-WAR-1AL*	*QWAR.zaas-1AL*	1A	473.6	tggtcgcaaaaatctccattca	tggtcgcaaaaatctccattcg	tgaggagctgtcaacaaaca

SSV, SDS sedimentation volume; TW, Test weight; WAR, Water absorbing rate.

**Table 5:** The KASP markers validated in the diverse panel.

Marker	QTL	Genotype	Frequency (%)	Phenotype	*P* value
*Kasp-SSV-1AS*	*QSSV.zaas-1AS*	CC	63.4	SSV: 31.6 mL	<0.05*
		AA	36.6	SSV: 29.2 mL	
*Kasp-SSV-4AL*	*QSSV.zaas-4AL*	AA	57.7	SSV: 30.6 mL	<0.05*
		GG	17.1	SSV: 28.5 mL	
*Kasp-TW-1AL*	*QTW.zaas-1AL*	CC	51.2	TW: 799.1 g/L	<0.05*
		TT	48.8	TW: 810.7 g/L	
*Kasp-TW-7BL*	*QTW.zaas-7BL*	AA	29.3	TW: 809.8 g/L	<0.05*
		GG	45.5	TW: 797.9 g/L	
*Kasp-WAR-1AL*	*QWAR.zaas-1AL*	AA	52.0	WAR: 52.0%	<0.05*
		GG	47.2	WAR: 61.0%	

SSV, SDS sedimentation volume; TW, Test weight; WAR, Water absorbing rate.

## Discussion

While yield improvement remains a primary goal in wheat breeding, enhancing processing quality has emerged as a major objective in modern breeding programs (He et al., 2011; Liu et al., 2025; Zhang et al., 2025). Although influenced by environmental factors, agronomic practices, and soil fertility, the substantial variation in processing quality is largely governed by genetic differences. Key quality parameters, SSV, TW, and WAR), are critical determinants of end-use quality (Arruda et al., 2016; Sallam et al., 2020; Miao et al., 2022; Aoun et al., 2022; López-Fernández et al., 2024; Vishwakarma et al., 2024). Therefore, identifying key genetic loci, developing breeder-friendly markers, and creating novel germplasm are essential for the sustainable production of high-quality wheat. In this study, we performed a GWAS for SSV, TW, and WAR using a panel of 310 wheat accessions. Our analysis identified stable and significant loci and facilitated the development of practical KASP markers, providing valuable genetic resources for molecular breeding aimed at improving wheat quality.

The extended LD inherent to elite wheat germplasm presents both an opportunity and a limitation for GWAS. Early studies in modern cultivars reported LD decay over 1–5 cM, translating to several megabases, which facilitates association detection with moderate marker density but limits mapping resolution (Breseghello and Sorrells, 2006). This extensive LD often results in broad association intervals encompassing numerous genes. The decay distance is highly population-dependent; landraces exhibit much faster LD decay (<1 cM) than modern breeding lines, allowing for finer mapping but requiring significantly higher marker density (Chao et al., 2010). With the advent of high-density SNP arrays and sequencing, studies have quantified LD more precisely, observing decay to an r² of 0.1 within approximately 3–5 Mb in diverse panels, setting a benchmark for required marker spacing (Voss-Fels et al., 2019). Furthermore, LD patterns are non-uniform across the genome, with generally slower decay in the A and B subgenomes compared to the D subgenome (Jordan et al., 2015). These factors collectively define the “GWAS threshold” for statistical significance and underscore that associated loci typically represent haplotype blocks rather than causal polymorphisms, necessitating downstream validation (Juliana et al., 2022).

### Comparison to previously reports

#### SDS sedimentation volume

SSV, TW and WAR are crucial traits governing wheat grain quality (Mohamed et al., 2022; Saini et al.,2022; Tian et al., 2022; Castellari et al., 2023; Li et al., 2025). As a key predictor of gluten quality, SSV has been extensively studied through QTL mapping, with numerous loci identified across the wheat genome (Zhou et al., 2021; Chang et al., 2011; Shvachko et al., 2024). Blanco et al. (1998) mapped 8 SSV QTL distributed on chromosomes 1AL, 1BS, 3AS, 3BL, 5AL, 6AL, and 7BS. Huang et al. (2006) have identified 3 SSV QTL on chromosomes 1B, 2D, and 5D, with 8.8-14.9% of the PVE in a DH population. Conti et al. (2011) detected 11 QTL for SSV located on chromosomes 1A, 1B, 3B, 4A, 4B, 6A, 6B, and 7A in the UC1113 × Kofa RIL population. Deng et al. (2015) reported 3 loci for SSV on chromosomes 1A, 1B, and 1D. Goel et al. (2019) have identified 5 SSV QTL accounting for 9.0-16.8% of the PVE on chromosomes 1B, 1D, 4A, 4B, and 7A in WL711/C306 RIL population. Rapp et al. (2019) mapped 5 novel loci for GPC and SSV in durum wheat, mainly distributed on chromosomes 1A, 2A, 3B, and 4D. Ruan et al. (2020) have detected 6 SSV loci on chromosomes 1A, 1B, 2B, and 3A in DH population. Semagn et al. (2021) have reported 17 SSV loci across four RIL populations, and mainly located on chromosomes 1A, 1B, 1D, 2D, 3A, 4A, 5A, 5B, 5D, and 6B. Chang et al. (2022) mapped major QTL for SSV, including *QSSV-1A*, *QSSV-1B.1*, and *QSSV-5D*, explaining 6.58%-15.53% of the PVE, respectively. Notably, the genes *Glu-A1*, *Glu-B1*, and *Pina-D1* were located within these three QTL regions and were considered to significantly influence SSV (Guzmán et al., 2022). In addition, Li et al. (2025) reported 8 QTL for SSV on chromosomes 1A, 2B, 2D, 5A, and 5B. Among them, *QSSV.sau-1A.1* was identified as a major and stable QTL, located at 510.3-531.2 Mb on chromosome 1A. In this study, we identified three loci for SSV, e.g. *QSSV.zaas-1AS* on chromosome 1A (26.4-27.9 Mb), *QSSV.zaas-4AS* on chromosome 4A (85.8 Mb), and *QSSV.zaas-4AL* on chromosome 4A (578.8-581.8 Mb). Among these, *QSSV.zaas-4AL* (578.8-581.8 Mb) was nearly with previously reported loci on chromosome 4AL (576.3-588.9 Mb) (Conti et al., 2011; Semagn et al., 2021). In contrast, no overlapping loci were found for *QSSV.zaas-1AS* and *QSSV.zaas-4AS*, suggesting they may represent novel loci of SSV (**Supplementary Table S7**).

#### Test weight

TW, or specific weight, is a key quality parameter defined as the weight of a known volume of grain. It serves as a vital indicator of grain quality, as low TW values often reflect poor grain filling, misshapen grains, or elevated moisture content. TW is mainly influenced by grain weight, shape, and volume, allowing this trait to be dissected into its constitutive components for analysis as individual yield-related factors (Corsi et al., 2021). Given its significant impact on flour extraction rates, extensive genetic mapping studies have been conducted to elucidate the genetic basis of TW. Multiple significant loci for TW have been identified on chromosomes 1A, 1D, 2A, 2B, 3B, 4B, 5D, 6B, and 7B (Narasimhamoorthy et al., 2006; Cabral et al., 2018). White et al. (2022) reported 5 TW loci on chromosomes 1B and 3B via GWAS in 150 cultivars. In this study, we identified 7 loci for TW, *QTW.zaas-1AS* (26.0 Mb), *QTW.zaas-1AL* (582.0 Mb), *QTW.zaas-4AL* (578.8-581.8 Mb), *QTW.zaas-4AL* (678.5-685.7 Mb), *QTW.zaas-5AL* (554.1-569.7 Mb), *QTW.zaas-6DS* (180.1 Mb), and *QTW.zaas-7BL* (493.2-498.7 Mb). Among these, *QTW.zaas-1AS* and *QTW.zaas-7BL* were nearly with previously reported loci on chromosomes 1A and 7BL (Narasimhamoorthy et al., 2006; Cabral et al., 2018). Conversely, no proximal or overlapping loci were observed for *QTW.zaas-1AL*, *QTW.zaas-4AL*, *QTW.zaas-5AL*, and *QTW.zaas-6DS*, indicating these may be novel loci.

#### Water absorption

WAR plays a crucial role in for wheat grain quality. High WAR can increase bread yield per unit of flour and improve softness. Genetic studies revealed that WAR controlled by multiple minor genes and with over 30 loci distributed across the whole genome (Lou et al., 2021; Wang et al., 2021; Gaur et al., 2022; Rahimi et al., 2023; Wondifraw et al., 2024). Zhao et al. (2024) reported 21 QTL for WAR on chromosomes 1B, 1D, 2A, 2D, 3A, 3B, 3D, 5B, 5D, 6B, 6D, and 7B. Among these, 6 QTL on chromosome 3A collectively 23.52% of the PVE. Four QTL were identified at 15.57 Mb, 152.26 Mb, 196.57-198.13 Mb, and 333.49 Mb of chromosome 6D, and explaining 3.93-4.25% of the PVE. Additionally, *qWA-5B.1* and *qWA-5B.2* on chromosome 5B with 3.72-4.27% of the PVE, and *qWA-3D* (*AX-108907834*) on chromosome 3D (23.0 Mb) with 7.51% of the PVE. Jin et al. (2016) reported 13 QTL for WAR on chromosomes 1A, 2B, 4A, 4B, 5D, 6A, 6B, 7A, 7B, and 7D. In this study, we identified 5 loci for WAR: *QWAR.zaas-1AL* on chromosome 1A (477.4 Mb), *QWAR.zaas-3BL* on chromosome 3B (454.1 Mb), *QWAR.zaas-4BL* on chromosome 4B (623.8 Mb), *QWAR.zaas-4DS* on chromosome 4DS1 (38.4 Mb) and *QWAR.zaas-4DS* on chromosome 4DS2 (208.5 Mb). Among these, *QWAR.zaas-1AL* and *QWAR.zaas-3BL* were nearly or overlapped with previously reported loci on chromosomes 1A and 3B (Jin et al., 2016; Zhao et al., 2024). In contrast, no nearly or overlapping loci were found for *QWAR.zaas-4BL*, *QWAR.zaas-4DS1* and *QWAR.zaas-4DS2*.

### Candidate gene analysis

Candidate gene analysis has further deepened the understanding of the genetic basis of SSV, TW and WAR. For SSV, MADS-box transcription factor (*TraesCS1A01G044900*) is postulated to be involved in a key developmental switch. MADS-box transcription factors are master regulators of wheat grain development. They coordinate a gene network controlling starch biosynthesis and storage protein synthesis, directly influencing grain filling and protein composition. By integrating hormone signals, they ultimately determine key yield and quality traits such as grain weight and gluten properties, making them crucial genetic targets for quality improvement in breeding programs (Raza et al., 2022; Zhang et al., 2024). The β-1,3-galactosyl transferase-like protein (*TraesCS1A01G047000*) could participate in gluten matrix assembly through physical or signaling interactions (Narciso et al., 2021; Cabas-Lühmann et al., 2024). Furthermore, E3 ubiquitin ligase (*TraesCS4A01G083500*) introduces a protein homeostasis mechanism, potentially targeting specific glutenin subunit precursors for degradation (Parveen et al., 2021; Lv et al., 2022; Ko et al., 2023). In addition, zinc finger protein (*TraesCS4A01G267600*) points to a connection between chloroplast function and grain protein quality, likely via the regulation of source-sink relationships (Rathan et al., 2022; Jaiswal et al., 2024; Manser et al., 2024).

In the case of WAR, several candidate genes highlight distinct physiological pathways. Auxin influx transporter (*TraesCS1A01G278400*) could potentially influence water absorption by modulating auxin distribution in the endosperm, thereby increasing internal grain porosity (Kabir et al., 2021; Li et al., 2021). Meanwhile, β-glucosidase (*TraesCS1A01G279000*) maybe facilitates water penetration by hydrolyzing β-1,4-glycosidic bonds in endosperm cell walls, thereby loosening the wall structure (Acin-Albiac et al., 2021; Zheng et al., 2021; Sun et al., 2025). MYB transcription factor (*TraesCS3B01G281500*) are key regulators influencing wheat grain WAR by modulating endosperm composition. They directly control the expression of genes responsible for storage proteins (e.g., glutenins) and cell wall polysaccharides like arabinoxylans. Both components are critical for forming the protein matrix and hydrophilic network in flour, which fundamentally determine its water-binding capacity and dough hydration properties. Therefore, allelic variation in specific MYB genes, potentially identified through GWAS, could be a major genetic determinant of WAR, offering targets for molecular breeding to improve wheat processing quality (Gao et al., 2021; Li et al., 2025). ABC transporter (*TraesCS4B01G331400*) may influence hydration by mediating ABA transport and aquaporin expression (Kumar et al., 2024). In addition, a zinc finger protein (*TraesCS4D01G062600*) and an E3 ubiquitin ligase (*TraesCS4D01G155700*) further contribute to WAR regulation, with the latter potentially modulating key enzymes involved in starch and cell wall metabolism via ubiquitination.

For TW, ethylene-responsive transcription factors (*TraesCS4A01G412200*, *TraesCS5A01G371300*, *TraesCS7B01G272300)* are critical regulators linking ethylene signaling to TW. They modulate the expression of genes involved in grain filling, nutrient remobilization, and maturation processes. By orchestrating the timing and efficiency of starch and protein accumulation in the endosperm, ERFs directly influence kernel plumpness and density, the core determinants of TW. Therefore, genetic variation in key ERF genes can impact final grain weight and quality, making them promising targets for breeding programs aimed at optimizing both yield and processing quality in wheat (Xu et al., 2022; Li et al., 2023; Shaw et al., 2023). Serine/threonine-protein kinases (*TraesCS4D01G059300*, *TraesCS5A01G350800*) likely regulate starch deposition through phosphorylation of enzymes in the sucrose-starch conversion pathway (Zhao et al., 2023; Alqudah et al., 2025). Dysregulation can result in chalky endosperm and lower test weight. Another ABC transporter (*TraesCS7B01G271500*) might be associated with enhance “sink strength” by actively transporting photosynthetic assimilates into endosperm cells, thereby improving grain plumpness and test weight.

In this study, candidate genes were preliminarily screened through bioinformatic annotation and expression profiling analyses. These candidates currently serve only as reference targets, as their biological functions remain to be experimentally validated. To systematically characterize these genes, the following research pipeline will be applied: (1) construction of a secondary mapping population coupled with KASP marker development for high-resolution genetic mapping; (2) comprehensive identification of target genes through integrated transcriptomic and genomic variation analyses; (3) functional validation using gene editing (e.g., CRISPR/Cas9) and transgenic complementation approaches.

### Potential implications in wheat breeding

Wheat processing quality is a typical polygenic trait that is challenging and costly to phenotype, making MAS an essential strategy for breeding improvement (Kumar et al., 2024; Yang and Song, 2024; Rasheed et al., 2025). While traditional breeding has been constrained by limited marker density and throughput, KASP offers a high-throughput, cost-effective, and accurate alternative approach (Rasheed et al., 2016; Kaur et al., 2020; Liu et al., 2023). KASP enables fluorescence-based genotyping of thousands of samples without requiring sophisticated instrumentation, making it particularly suitable for large-scale breeding programs. Its key advantages include: (1) flexible primer design based on functional SNPs; (2) high genotyping accuracy (>99%), including reliable heterozygote detection; and (3) low cost per data point (approximately $0.1-0.3) (Rasheed et al., 2016; Kaur et al., 2020; Liu et al., 2023). KASP markers have already been successfully deployed in wheat quality breeding for major loci such as *Glu-D1d* (glutenin subunit) and *Pina/Pinb* (grain hardness). In this study, we developed 5 KASP markers for stable loci, *Kasp-SSV-1AS*, *Kasp-SSV-4AL*, *Kasp-TW-1AL*, *Kasp-TW-7BL*, and *Kasp-WAR-1AL*. These markers provide reliable, breeder-friendly tools for quality-oriented selection. Their implementation facilitates early identification of favorable alleles, reduces phenotyping costs, and accelerates cultivar development. Furthermore, elite accessions carrying favorable alleles could be as valuable parental resources for improving wheat processing quality.

We have calculated the total PVE by the significant loci for each trait. The stable MTAs for SSV, TW, and WAR collectively 22.7-27.0%, 58.2-77.2%, and 35.7-40.5% of the PVE, respectively, confirming their major effects. Based on the PVE and multi-environment stability, we propose the following marker deployment strategy to guide breeding: Marker Priority: The locus on chromosome 1AL (*QTW.zaas-1AL* & *QWAR.zaas-1AL*) is the highest priority due to its exceptionally large and stable effect on both TW and WAR. Optimal Allele Combinations: Considering that pyramiding 2–3 major loci is highly effective in wheat breeding, we simulated the value of combining our validated KASP markers. The most promising haplotype combination is *QSSV.zaas-1AS* & *QTW.zaas-1AL* & *QWAR.zaas-1AL*, followed by the combination *QSSV.zaas-1AS*&*QTW.zaas-1AL*&*QTW.zaas-7BL*, *QSSV.zaas-4AL*&*QTW.zaas-1AL*&*QWAR.zaas-1AL*, and *QSSV.zaas-4AL*&*QTW.zaas-1AL*&*QTW.zaas-7BL*.

## Future prospects for high-quality wheat breeding

Also, this study has several limitations. First, the GWAS was performed using a panel of 310 wheat varieties from the HHWWR. Although this panel was carefully selected to represent the genetic diversity of China largest commercial wheat production region, the sample size remains moderate for dissecting complex polygenic traits such as SSD, TW, and WAR. In addition, the panel mainly represents the Huang-Huai region and lacks materials from other major ecological wheat zones, which may limit the generalizability of the identified markers across different genetic backgrounds. To partially mitigate the constraints of sample size, we employed a high-density SNP set and a MLM model that accounted for population structure and kinship, which helps improve the reliability of the associations. Second, the candidate genes proposed in this study were inferred based on genomic annotations and orthologs involved in grain quality and grain filling; however, these statistical associations have not yet been functionally validated. Complementary evidence from transcriptomic, physiological, or transgenic studies is required to confirm their causal roles. Third, given that standard multiple-testing corrections are often overly conservative for complex traits, a suggestive significance threshold was adopted-an approach also used in other crop GWAS studies. While this facilitated the detection of loci with moderate effects, it underscores the polygenic architecture of grain quality traits and highlights the need for further validation. Future work will focus on expanding the germplasm collection, functionally characterizing candidate genes, and validating the pleiotropic loci in diverse genetic backgrounds to advance the breeding of high-quality wheat.

In recent years, wheat breeding objectives have progressively shifted from a singular focus on yield toward a dual emphasis on both yield and quality improvement. SSV, WAR, and TW have become core selection criteria in breeding programs. Variation in SSV is primarily attributed to differences in HMW-GS composition, and future MAS or gene editing technologies are expected to enable precise regulation of specific glutenin subunit expression (Rasheed et al., 2025). WAR is strongly associated with starch physicochemical properties, and current research is increasingly focused on elucidating the genetic regulation of starch biosynthesis pathways. Although the genetic basis of TW is relatively well characterized, this trait remains highly sensitive to post-anthesis environmental conditions such as temperature and precipitation. Therefore, it is necessary to integrate stress resilience and quality traits into a unified selection framework. Developing multi-trait genomic selection models, combined with phenomics and genomics, and integrated with gene editing technologies, will be central to future breakthroughs in high-quality wheat breeding.

## Conclusions

In summary, we have identified 15 stable QTL, including three for SSV, five for WAR, and seven for TW. Among these, 10 represent potentially novel loci, whereas the other 5 loci overlapped with previous reported genes or loci. Candidate gene analysis linked these genomic regions to biological processes such as lipid metabolism, signal transduction, and cell wall modification. Furthermore, we developed and validated five breeder-friendly KASP markers. These findings enhance our understanding of the genetic architecture underlying wheat quality and provide practical molecular tools for breeding selection, paving the way for the development of superior wheat varieties with improved end-use properties.

## Data Availability

The datasets presented in this study can be found in online repositories. The names of the repository/repositories and accession number(s) can be found in the article/**Supplementary Material**.

## Supplementary material

Supplementary Table 1 The details of the SSV, WAR and TW for the 310 accessions.

Supplementary Table 2 The correlation matrix for the SSV, WAR and TW in the diverse panel.

Supplementary Table 3 ANOVA of SSV, WAR and TW for the diverse panel across all the environments.

Supplementary Table 4 The SNP genotyping details of the 310 wheat accessions by 120K SNP array.

Supplementary Table 5 The genes list located in the confidence interval by annotation.

Supplementary Table 6 The reported loci for SSV, TW and WAR in previous studies.

Supplementary Table 7 The KASP markers validated in the diverse panel.
