# Supplementary material for: Uncover the genetic basis of processing quality related traits in common wheat (Triticum aestivum L.) using genome-wide association mapping
Source: Front Plant Sci. 2026 Mar 3;17:1755182. doi: 10.3389/fpls.2026.1755182 (PMC12992234; doi:10.3389/fpls.2026.1755182)
Supplement: Supplementary Table 2 — The correlation matrix for the SSV, WAR and TW in the diverse panel. [file Table2.docx]

Table S2 The correlation matrix for the SSV, WAR and TW in the diverse panel

| Trait | SSV | WAR |
| --- | --- | --- |
| WAR | 0.39 |  |
| TW | 0.28 | 0.15 |

SDS sedimentation volume (SSV), test weight (TW), and water absorption rate (WAR)
