# Supplementary material for: Uncover the genetic basis of processing quality related traits in common wheat (Triticum aestivum L.) using genome-wide association mapping
Source: Front Plant Sci. 2026 Mar 3;17:1755182. doi: 10.3389/fpls.2026.1755182 (PMC12992234; doi:10.3389/fpls.2026.1755182)
Supplement: Supplementary Table 3 — ANOVA of SSV, WAR and TW for the diverse panel across all the environments. [file Table3.docx]

**Table S2 ANOVA of SSV, WAR and TW for the diverse panel across all the environments**

| Source of variation | Df | Significance level (*F*-value) | | |
| --- | --- | --- | --- | --- |
|  |  | **SSV** | **WAR** | **TW** |
| Genotypes | 309 | *** | *** | *** |
| Environments | 3 | *** | *** | *** |
| Replicates (environments) | 2 | *** | *** | *** |
| Genotype*Environment | 927 | *** | *** | *** |
| Error | 2472 |  |  |  |

SSV, SDS sedimentation volume; TW, Test weight; WAR, Water absorbing rate
