# Supplementary material for: Uncover the genetic basis of processing quality related traits in common wheat (Triticum aestivum L.) using genome-wide association mapping
Source: Front Plant Sci. 2026 Mar 3;17:1755182. doi: 10.3389/fpls.2026.1755182 (PMC12992234; doi:10.3389/fpls.2026.1755182)
Supplement: Supplementary Table 4 — The SNP genotyping details of the 310 wheat accessions by 120K SNP array. [file Table4.docx]

**Table S3 The SNP genotyping details of the 310 common wheat accessions by 100K SNP array**

| Chromosome | No. of the marker | Physical length (Mb) | Marker density (marker/Mb) |
| --- | --- | --- | --- |
| 1A | 5156 | 598.6 | 8.6 |
| 2A | 6537 | 787.7 | 8.3 |
| 3A | 5606 | 754.0 | 7.4 |
| 4A | 5297 | 754.2 | 7.0 |
| 5A | 5733 | 713.3 | 8.0 |
| 6A | 4905 | 622.6 | 7.9 |
| 7A | 5676 | 744.5 | 7.6 |
| 1B | 4777 | 700.3 | 6.8 |
| 2B | 6541 | 812.7 | 8.0 |
| 3B | 6173 | 851.9 | 7.2 |
| 4B | 4659 | 673.6 | 6.9 |
| 5B | 5908 | 714.8 | 8.3 |
| 6B | 5284 | 731.1 | 7.2 |
| 7B | 5581 | 763.7 | 7.3 |
| 1D | 3931 | 498.6 | 7.9 |
| 2D | 5270 | 656.4 | 8.0 |
| 3D | 4703 | 619.5 | 7.6 |
| 4D | 3568 | 518.2 | 6.9 |
| 5D | 4755 | 569.8 | 8.3 |
| 6D | 3725 | 495.3 | 7.5 |
| 7D | 5051 | 642.8 | 7.9 |
| A | 38910 | 4974.7 | 7.8 |
| B | 38923 | 5248.0 | 7.4 |
| D | 31003 | 4000.6 | 7.7 |
| ALL | 108836 | 14223.3 | 7.7 |
